# Virtual Body Ownership Illusions for Mental Health: A Narrative Review

**DOI:** 10.3390/jcm10010139

**Published:** 2021-01-03

**Authors:** Marta Matamala-Gomez, Antonella Maselli, Clelia Malighetti, Olivia Realdon, Fabrizia Mantovani, Giuseppe Riva

**Affiliations:** 1 Center for Studies in Communication Sciences “Luigi Anolli” (CESCOM), Department of Human Sciences for Education “Riccardo Massa”, University of Milano-Bicocca, 1-20126 Milan, Italy; olivia.realdon@unimib.it (O.R.); fabrizia.mantovani@unimib.it (F.M.); 2 Institute of Cognitive Sciences and Technologies (ISTC), National Research Council (CNR), 44-00185 Rome, Italy; antonella.maselli@istc.cnr.it; 3 Department of Psychology, Catholic University of Milan, 1-20123 Milan, Italy; clelia.malighetti@unicatt.it (C.M.); giuseppe.riva@unicatt.it (G.R.); 4 Applied Technology for Neuro-Psychology Laboratory, Istituto Auxologico Italiano, IRCCS, 1-20149 Milan, Italy

**Keywords:** virtual reality, body illusions, embodiment, mental health

## Abstract

Over the last 20 years, virtual reality (VR) has been widely used to promote mental health in populations presenting different clinical conditions. Mental health does not refer only to the absence of psychiatric disorders but to the absence of a wide range of clinical conditions that influence people’s general and social well-being such as chronic pain, neurological disorders that lead to motor o perceptual impairments, psychological disorders that alter behaviour and social cognition, or physical conditions like eating disorders or present in amputees. It is known that an accurate perception of oneself and of the surrounding environment are both key elements to enjoy mental health and well-being, and that both can be distorted in patients suffering from the clinical conditions mentioned above. In the past few years, multiple studies have shown the effectiveness of VR to modulate such perceptual distortions of oneself and of the surrounding environment through virtual body ownership illusions. This narrative review aims to review clinical studies that have explored the manipulation of embodied virtual bodies in VR for improving mental health, and to discuss the current state of the art and the challenges for future research in the context of clinical care.

## 1. Introduction

The last two decades have seen how immersive virtual reality (VR) systems have become powerful tools to induce body ownership illusions in both healthy and clinical populations [1,2]. VR has been described as a technology that simulates ‘reality’ [2,3]. In immersive VR, computer-generated simulations control hardware devices that can stimulates our sensory organs as it happens in the physical world. On the one hand, “anything that can happen in reality can be programmed to happen but ‘virtually’ [2], and on the other VR also offers an infinity of possibilities that you don’t have in the physical world.” [4,5]. The application of VR as a non-invasive technology to induce virtual body ownership illusions specifically in clinical populations has been explored especially in the last ten years [6,7,8]. One of the most important advantages of using immersive VR in clinical settings is that immersive VR environments allow researchers or clinicians to manipulate the multimodal stimuli inputs, thus allowing the patients to feel ‘present’ in the displayed environment [9,10].

Another important factor that makes VR an effective tool to be used in clinical populations is that through the use of virtual avatars and by applying synchronous multisensory correlations (see Body Illusions below) it is possible to induce the sense of embodying a virtual body. A large number of studies have demonstrated that through virtual embodiment in immersive VR it is possible to experience the sense of ownership over a virtual limb [11] and even over an entire virtual body [12]. Moreover, VR allows the experimenter to manipulate not only the virtual environment but also the embodied virtual body, providing them with specific features that would otherwise be impossible in physical reality [10]. For this reason, VR systems have many potential applications in the fields of psychotherapy, rehabilitation, and behavioral neuroscience (see [13,14] for a review). This narrative review aims to review clinical studies that have explored the use of virtual body ownership illusions in immersive VR for different clinical applications. Moreover, we discuss the advantages of using such virtual BOIs to modulate the internal body representation, and the limitations arising from the difficulty to modify the inner—as opposed to outer—body perception, which is an aspect from which clinical populations could benefit.

### 1.1. Alterations in Body Representation in Pathological Conditions

The accurate perception of oneself and of the surrounding environment are both key elements to enjoy mental health and well-being [15]. Suffering from a mental or neurological disorder can lead to a distortion of such perception. For instance, alterations in body representation have been reported in patients with eating disorders, chronic pain [16], or psychiatric pathologies such as schizophrenia [17], among others. Some consequences of experiencing body representation alterations include a negative perception of the body and body dissatisfaction in patients with eating disorders [18,19], changes in the perception of the size of the painful limb in patients with complex regional pain syndrome [20] or with hand osteoarthritis [21], phantom limb sensations in amputees (the persistent feeling that the amputated limb is still there) [22], or disturbed sensations of limb ownership like somatoparaphrenia and asomatognosia (delusional beliefs typically observed in patients with right hemisphere brain damage) (see [23,24]).

Interestingly, altered body perceptions analogous to those reported in pathological conditions can be induced in healthy individuals using controlled experimental paradigms. All experimental manipulations that induce body illusions (BIs) rely on exposing participants to multisensory conflicts (see Body illusions below for a detailed explanation). Experimentally induced BIs have brought tremendous insights into the nature of self-perception, showing how self-body perception is a dynamic and extremely flexible emerging percept. BI studies have also revealed how altered body-perception states can have a significant impact on perception, behaviour, and cognition, particularly in the case of body ownership illusions (BOIs). While experiencing a BOI over an artificial object (e.g., a rubber or a virtual hand), the physiological state of the body (e.g., the body temperature) [25,26] and the neurophysiological response to external stimuli (e.g., tactile, thermal, or noxious stimuli) [27,28,29] are temporarily altered, and the temporal constraints for the integration of independent bodily stimuli get looser [30]. Such different effects can be explained with the concept of “body matrix”, which illustrates a direct interrelationship between the cognitive representation of the body (e.g., body ownership) and homeostatic functions (e.g., thermoregulation) [31].

### 1.2. The Concept of “Body Matrix”

The concept of a cortical “body matrix” was introduced by Moseley and colleagues in 2012 [31]. It was described as a dynamic and flexible multisensory representation of the space directly surrounding the body, aligned with a body-centered frame of reference. Such body representation results from the processing of sensory signals by different brain areas, e.g., visual, tactile, and proprioceptive, and the further integration of these signals in higher associative brain areas [31]. Hence, the body matrix is considered a high-level representation of the self in the environment, which interprets and integrates different bodily signals and sensory stimuli from the directly surrounding environment into a coherent body-centered framework. Therefore, the body matrix defines a self-body model that allows the top-down integration of sensory processing by integrating the incoming (bottom-up) sensory signals, which then drives changes in the body behaviour during the interaction with the surrounding environment [1,32]. The body matrix in essence is conceived of as a prerequisite that allows for efficient self-regulation and adaptaion to the dynamic environment, and as such, it allows for the adaptation to profound changes in the anatomical, postural, and conceptual (believes) representations of our own body [31,33].

The body matrix can be seen as a functional construct that maintains the body’s integrity at both the homeostatic and psychological levels, and that adapts the body representation to changes in our body structure and body orientation in the surrounded environment (from both ontogenetic and phylogenetic points of view) see [30]. In contrast with other authors who defend a dichotomous representation of the body in the brain as a posture-sensorimotor ‘body schema’ and a conscious-evaluative ‘body image’ [34], the body matrix proposes an interrelationship between the cognitive representation of the body (e.g., body ownership, body memories) and the homeostatic body functions (e.g., thermoregulation) in a single representation; that is, one body in the brain [31].

A large body of evidence from research experiments using BIs to modulate body representations have shown that it is possible to modulate the internal body representation through ad-hoc controlled experimental manipulations. If we consider one step further, the concept of body matrix entails the idea that through the use of BIs, it is possible to modulate this internal body representation when it has been distorted due to a clinical condition (e.g., chronic pain, eating disorder, motor disorder, etc.) in order to recover its functional structure [1,31].

### 1.3. Body Illusions to Manipulate the Body Matrix/Body Representation

BIs refer to altered perceptual states where the perception of the self-body significantly deviates from the physical body’s configuration, for example in aspects like perceived size, shape, posture, location, or sense of ownership [35]. Different established experimental paradigms allow to temporarily induce such altered perceptual states in a predictable and systematic manner in healthy participants. These paradigms are based on ad-hoc manipulations of bodily stimuli so that, by delivering conflicting information about the body, illusory experiences arise as the brain “looks for” an explanatory solution to the conflict [36]. For example, in order to explain the sensation that one keeps touching the tip of her nose while feeling that her elbow is extending, the illusory sensation of an elongating nose arises [37].

BIs can be triggered through relatively simple experimental manipulations (e.g., through visuo-tactile or visuo-motor correlations), supporting the overall view that self-body perception is built dynamically on the base of multisensory integration processes and of the prior knowledge we have about the human body. Hence, even though we may have the notion that our own internal body representation is stable, it is, in truth, highly malleable. Through BIs, subjects can embody fake body parts or whole fake bodies, which are perceived as belonging to or substituting their physical body. The sense of embodiment has been described as the sense of one’s own body [38], embedding three components: (1) body ownership (owning the physical body), (2) self-location (perceived location of the self in space), and the (3) sense of agency (controlling motor intentions and actions of the physical body) [18,39]. Even though there are different types of BIs that manipulate the body representation, such as kinesthetic or body distortion illusions [40] and out-of-body experience illusions [41,42], in this narrative review we will focus on body ownership illusions. The experimental paradigms for eliciting BOI all rely on establishing a “connection” between the real body (part) and a fake bodily shaped object via multisensory stimulation.

The first formal study to investigate BOIs was conducted by Tastevin at 1937 [43], who described how people could perceive an artificial finger protruding from a cloth as their own finger, when the latter was hidden from view. Then, Botvinick and Cohen (1998) [44], presented the Rubber Hand Illusion (RHI) paradigm, giving rise to a rich line of research on the topic. In the RHI, participants are presented with a visible rubber hand placed close to their occluded real hand, and the two hands—rubber and real—are stroked synchronously, which elicits the illusory experience that the touch is felt on the rubber hand. An associated recalibration of the perceived position of the real hand towards the rubber hand, known as the proprioceptive drift, was also reported. This study was soon followed by numerous other experimental works that adopted and modified the RHI paradigm to investigate the perceptual, cognitive, and neurophysiological underpinnings of the sense of body ownership. As for the RHI, full-body ownership illusions can be achieved through visuo-tactile stimulation [45,46,47], visuo-motor correlations [48,49], as well as through static visuo-proprioceptive sensory signals [50]. By means of BOIs it is possible to induce the sense of body ownership either over a fake body part or over a full fake body.

BOIs have been proposed to arise as the result of an inference process in which the brain attributes all the incoming sensory information about the body (visual information from the fake body and somatosensory sensations from the real body) to the same common cause, which is the own body [35,51]. By giving access to a controlled manipulation of the internal body representation, BOIs have been established as an effective tool to modulate the distorted internal body representation in populations suffering from different clinical conditions [31,52].

### 1.4. VR to Induce BOIs in Clinical Populations

The development of new technologies such as VR systems has allowed researchers and clinicians to induce BOIs over virtual avatars. The appearance of the virtual avatar can be shaped according to the desired morphological characteristics. Also, new motion capture systems allow to replicate the participant’s movements on the avatar with high fidelity. This results in a more natural BOI from an anthropomorphic point of view. One of the first studies to replicate the RHI in virtual reality was the virtual hand illusion study conducted by Slater and co-authors in 2009 [8]. In their study, the authors applied synchronous visuo-tactile stimulation (VTS) to an embodied virtual arm observed from a first-person perspective (1PP) and to the participant’s real arm, which resulted in the participants reporting feelings of ownership over the observed virtual arm [11]. Moreover, the virtual hand illusion can also be induced by means of synchronous visuo-motor correlations (VMC) [53]. Immersive devices, like head-mounted displays that occlude the view of the real body, allowed to extend ownership illusions toward an entire fake body, e.g., a mannequin or a virtual body [47]. In these studies, participants could see a full-sized humanoid body from a 1PP and, when receiving synchronous visuo-tactile stimulation, they had the perceptual illusion that the virtual body was indeed their own, and showed a distressing reaction when the artificial body was threatened. Later studies further showed that full-BOIs over a virtual body could also be induced by just observing full virtual bodies from a 1PP in spatial overlap with the real body (i.e., through visuo-proprioceptive congruent cues) [50], which could be made even more vivid through synchronous VMC between the virtual and the real bodies [12].

Virtual BOIs have been recently investigated in clinical populations to modulate their distorted body representation. The possibility to adapt the virtual body representation to the specific characteristics required by researchers or clinicians makes virtual BOIs a powerful tool to study and modulate altered body representations. In this regard, several studies have shown that through the use of humanoid virtual BOIs, people can experience ownership over virtual bodies of a different gender [49], age [54,55] or skin colors [56,57] having a different posture [56,58,59], an altered size of some body parts [45,60,61], or a certain degree of transparency of the virtual body [62]. As we review below, such changes in body representation, possible through virtual BOIs, not only modulate internal body representations but can also affect participants’ behavioral and physiological responses such as their perception of pain [63], motor learning [64], and cognitive and responses [65,66]. Based on these findings, virtual BOIs have been shown to have many potential applications in the fields.

## 2. Search Strategy

Bibliographical data was collected on 18 April 2020, by using the following bibliographic databases: PubMed, EMBASE, Scopus, Web of Science, and PsycINFO. For each database, we used the following combination of research keywords: (1) “mental health” OR “mental disorder” OR “mental illness” OR “mental disease” AND “body illusions”; (4) “mental health” OR “mental disorder” OR “mental illness” OR “mental disease” AND “virtual reality”; (5) “mental health” OR “mental disorder” OR “mental illness” OR “mental disease” AND “virtual embodiment”; (6) “mental health” OR “mental disorder” OR “mental illness” OR “mental disease” AND “body ownership illusion”; (7) “mental health” OR “mental disorder” OR “mental illness” OR “mental disease” AND “body-swapping”. Only English full-text available articles were included in our research (conference paper and review articles were excluded), studies citation were retrieved independently for each string of keywords across all databases. The first list of the collected studies during the bibliographic research was exported to Mendeley to remove duplicated studies and then imported to Rayyan [67] for the title and abstract screening, indicating inclusion or exclusion criteria for study selection.

### Eligibility Criteria

The present review aims to discuss different research studies using virtual BOIs for changing body representation in clinical populations. Bibliographical research was limited to studies using humans and written in English. Further, the selected studies had to accomplish the following criteria:

(1) The studies must have been directed to improve mental health (e.g., pain perception, body representation, psychological or psychiatric conditions, and physiological response) in clinical populations. Interventions directed to investigate methodological aspects of how to induce virtual BOIs or to investigate technical aspects of virtual BOIs in clinical populations were excluded. (2) The studies must be directed to a group of clinical populations, between and within-group study design have been included. Single case or pilot studies with a tiny sample size were excluded as the results from such studies are not strong enough to show the effectiveness of virtual BOIS in improving mental health in clinical populations.

## 3. Virtual Body Ownership Illusions for Mental Health

### 3.1. Virtual BOIs to Modulate Pain Responses

The impact of body view on pain processing was known well before virtual BOIs were included in or studied as clinical treatments [68]. Research had shown that not only seeing the body can be analgesic, but also changing the appearance (e.g., the size or colour of the painful limb) could modulate this analgesic effect [56,69]. By giving the possibility to arbitrarily change the morphology and the visual appearance of the virtual body, virtual BOIs have been widely tested in people suffering from pain or chronic pain conditions, such as patients with complex regional pain syndrome (CRPS), patients with an amputated limb, patients with nerve injury, or patients suffering from a traumatic condition (e.g., osteoarthritis) [69]. One example is a study by Matamala-Gomez and colleagues (2019) [70], who investigated how altering the visual appearance of the painful virtual arm, in terms of size (big, normal, small) and transparency (0%, 25%, 50%, 75%) modulated pain perception in two different groups of chronic arm pain patients (CRPS and peripheral nerve injury (PNI)). Interestingly, in contrast to a study conducted on healthy subjects [59], Matamala-Gomez et al. (2019) [70] found that increasing the transparency of the observed virtual arm decreased pain ratings in patients with CRPS, but this did not occur in those with PNI. On the other hand, increasing the virtual arm size slightly increased pain ratings only in CRPS patients. Moreover, the exposure to all seven VR conditions globally decreased the mean pain ratings by half by the end of the experiment compared to the pain ratings at baseline. Furthermore, the authors found that patients with chronic pain can achieve ownership and agency levels over a virtual arm comparable to healthy participants, demonstrating that virtual BOIs can also be achieved in clinical populations that present body distortions.

Virtual BOIs over a virtual arm have also been tested in patients with hand osteoarthritis (HOA) who present body distortions because of the injury [21]. In this study, patients with painful HOA observed their most affected hand in and outside of a real-time mediated reality system, whereby they witnessed an illusory stretching of the hand and changes in sensory stimuli (visuo-tactile and proprioceptive signals). After the exposure, the authors assessed six statements relating to the emotional experience, perceived hand size, susceptibility, ownership and agency over the virtual arm in a 7-point Likert scale questionnaire, as well as the pain intensity. The results from this study showed that stretching the hand both inside and outside of the virtual environment led to a reduction in subjective pain ratings. Nevertheless, although virtual stretching led also to changes in body perception, it did not affect the pressure pain threshold.

The studies just mentioned support that visual manipulations of the body can modulate pain perception in clinical populations. These results are in line with two studies showing that observing a magnified body part increases the experimental heat pain thresholds in healthy subjects [71,72]. However, in both studies, a reduction of pain occurred only when applying synchronous multisensory correlations (i.e., visuo-tactile stimulation) between the real and the virtual limb. This is in agreement with another study in which patients with CRPS embodied a virtual arm and observed their affected virtual limb flashing in synchrony with their own detected heartbeat, or asynchronously in the control condition. The authors found that, in the synchronous heartbeat condition, CRPS patients reduced their pain ratings and improved their motor limb function relative to the asynchronous control condition and to a healthy control group [73]. In addition, in another study that attempted to modulate neuropathic pain in 20 patients with spinal cord injury, the authors showed that inducing virtual BOIs over virtual lower limbs together with synchronous tactile stimulation led to a mild analgesia, which was not the case for the asynchronous condition or in a healthy control group. These findings cannot be explained as a result of different ownership levels, since the authors did not find differences between groups in global body ownership as tested in the full body ownership illusion [74]. Likewise, patients with chronic pain who underwent a full-BOI over a virtual body, together with synchronous visuo-tactile stimulation, reduced their pain intensity ratings by 37% [75].

Another element that has been demonstrated to be effective in reducing pain perception when using BOIs is synchronous visuo-motor correlation. One of the most well-known pain relief applications of BOIs induced through synchronous VMC is mirror visual feedback therapy (MVFT). In the MVFT the patient’s healthy limb is reflected in a mirror and, seeming visually superimposed on the location of the affected limb, creates the illusion that the affected limb has recovered. Then, when patients move their healthy limb, they have the illusion of moving their affected limb. Such movement illusions result in pain relief in patients with phantom limb pain (see [76] for a review). Following this approach, some recent work has attempted to use virtual BOIs over virtual limbs to reduce pain perception in clinical populations. For instance, Osumi et al. (2019) [77] used a VR-MVFT system in amputee patients and in patients with brachial plexus avulsion injury with phantom limb pain. Their results showed more alleviation of the phantom limb pain in patients with brachial plexus avulsion injury compared with amputee patients. In addition, inducing a virtual arm illusion in augmented VR through synchronous VMC in amputee patients has also resulted effective in relieving phantom limb pain perception from pre-treatment to the last treatment session by 47% for weighted pain distribution, 32% for the numeric rating scale, and 51% for the pain rating index [78]. Pain relief was also found in a study by Alphonso et al. (2012) [79] when using the virtual arm illusion in amputee patients.

Some authors have opted for full-BOI rather than virtual BOI of limbs. In a study conducted by Hwang and collaborators [80], the authors compared the effect of performing three different condition on pain relief in patients with CPRS: (i) motor rehearsal in a swapped virtual body, (ii) mental rehearsal, or (iii) observed movement. Even though pain intensity did not decrease significantly after performing either of the three conditions, the body perception disturbance (i.e., the distortion in body representation associated with chronic severe pain) improved significantly after performing the virtual body swapping condition compared to the other conditions.

### 3.2. Virtual BOIs to Modulate Motor Responses

In the last years, BOIs have been tested as tools for neurorehabilitation [52], to regulate alterations of body representation in the brain and to restore motor ability. More specifically, it is possible to “trick” the brain through simple BOIs which may represent an effective strategy to boost and wake up the brain multisensory capabilities that can be latent or weakened after suffering a brain injury. According to this, the inter-sensory conflicts induced by the BOIs may facilitate the restoring of the altered sensory and motor representations related to body awareness disorders [52]. Currently, the most widely used BOI for motor recovery in patients with motor disorders is MVFT [76]. Nevertheless, even though the effectiveness of MVFT to modulate body representation and to improve motor recovery after stroke has been largely demonstrated [81,82,83,84], there are still some physical limitations to inducing a good enough sense of ownership and full embodiment over the observed body (part) in the mirror, which may weaken the rehabilitation outcome [59]. Hence, the development of VR systems through which it is possible to induce embodiment of a full virtual body observed from a first-person perspective [50], and therefore to manipulate morphological characteristics of the represented virtual body [48,56] as well as the visuo-motor mapping between the real and the virtual body, offers a potential powerful alternative to the traditional MVFT [85].

Weber and colleagues (2019) [86] examined the feasibility of an immersive VR mirror therapy for patients with stroke presenting upper limb paresis. In this study, ten patients with chronic stroke presenting upper limb paresis completed a 12-session program, 30 min per session, of immersive VR mirror therapy. The VR system induced the virtual arm illusion and provided virtual movement of the paretic upper limb while suppressing the visual representation of the non-paretic side, which resulted in a small improvement in upper limb motor recovery, but not significantly different from baseline [86]. Moreover, the virtual arm illusion has also been used for improving upper limb movements in a single patient suffering from Parkinson’s disease [87]. A VR rehabilitation program based on the MVFT has also been used to improve the postural balance and gait ability of patients with chronic stroke [88]. The results from this study demonstrated that virtual reality reflection therapy significantly improves postural balance and gait after four weeks of 30-min training sessions, five days a week [88]. VR systems are particularly important in the context of motor disorders because they are a potential tool to control and modulate on-line the motor behaviour of the user through the interplay between motor control loop mechanisms and the effects of embodiment that could drive and “attract” performed movement as in the “self-avatar follower effect” [89], the line-circle experiment [90].

### 3.3. Virtual BOIs to Modulate Psychological Responses

The use of virtual embodiment to provide virtual BOIs is also useful to modify and assess the experience of the body in patients with psychological or psychiatric disorders [91]. For instance, a recent study used a variant of the virtual hand illusion in patients with obesity and in healthy participants to assess multisensory integration processes [92]. The findings from this study demonstrate that, whereas patients affected by obesity had a typical subjective experience of the illusion with synchronous VTS, the proprioceptive drift was reduced compared to that of healthy subjects. Other researchers have used virtual-full-BOI to investigate whether ownership over a virtual body with a skinny abdomen might be successfully experienced in patients affected by obesity [93]. The authors showed that virtual-full BOI was induced in individuals with obesity to the same extent as it was in healthy-weight individuals. Both healthy-weight individuals and individuals with obesity showed a reduction of the proprioceptive drift error after synchronous VTS, but not after asynchronous VTS, with respect to the baseline. Moreover, Keizer and co-authors (2016) [94] also used virtual-full BOI induced by synchronous VTS to investigate whether size estimation of body parts that are more emotionally salient than the hand in patients with anorexia nervosa (AN) is altered compared with a healthy control group. The authors asked the patients to estimate their body size (shoulders, abdomen, hips) before the virtual-full BOI was induced, directly after induction and at ~2 h 45 min follow-up. The results showed that patients with AN decrease the overestimation of their shoulders, abdomen, and hips directly after the virtual-full BOI was induced. Furthermore, the improvements in body size estimation could still be observed in the AN group at the follow-up assessment time. However, the healthy control group also showed changes in body size estimation after the virtual-fullBOI, but the effect showed a different pattern than that of the AN group [94]. In contrast, a recent study showed that virtual-fullBOI, induced through synchronous VTS, did not reduce body image distortion in patients with AN [95]. In the study from Provenzano and co-authors, the authors evaluated body overestimation and dissatisfaction in 20 patients with AN by asking participants to choose the avatar that best resembled their real and ideal virtual body with the perceived/ideal body task. During the experimental session, participants were exposed to three embodiment blocks in which synchronous and asynchronous VTS was applied to three different bodies (the perceived body, −15% thinner body, +15% fatter body). After each embodiment block participants repeated the perceived/ideal body task to measure the effects of the embodiment of different sized avatars on body dissatisfaction [95]. The results from this study demonstrate that the desire of a thinner body induced a higher body dissatisfaction in AN. Moreover, the sense of embodiment toward the virtual body was stronger after synchronous VTS in both groups, but did not reduce body image distortion in participants with AN. Patients with AN reported more negative emotions after being embodied in the fattest avatar, which scaled with symptoms severity [95]. Finally, a recent case study using virtual-full BOI in patients with AN supports that virtual-full BOI can be employed to effectively assess changes in multisensory bodily integration and can act as a driver for these changes to improve body perception in patients with AN [48].

In addition to using virtual-full BOIs for improving body perception in patients presenting eating disorders, full BOIs in virtual reality have also been exploited for improving empathy or recalling traumatic memories. In a study conducted by Seinfeld and colleagues (2018) [46], the authors induced virtual-full BOI in intimate partner violence offenders to allow them to be in the body of a victim of domestic abuse. More specifically, a group of male domestic violence offenders and a control group of men without a history of violence experienced a virtual scene of abuse from a first-person perspective. During the VR experience the participants’ real bodies were replaced with a life-sized virtual female body that moved synchronously with their own real movements (synchronous VMC). Participants’ emotion recognition skills were assessed before and after the virtual experience. The authors observed that after being embodied in a female victim virtual body, offenders improved their ability to recognize fearful female faces and reduced their bias towards recognizing fearful faces as happy, thereby improving their empathic abilities [49]. In a different study, a virtual-full BOI over a virtual body with different morphological characteristics was induced in patients with depression to improve self-compassion [92]. The patients had to reproduce a compassionate speech from the perspective of an adult or a child virtual body. After three repetitions of the VR experience, there were significant reductions in depression severity and self-criticism, and a significant increase in self-compassion, from baseline to a 4-week follow-up [96]. Therefore, changing the morphological or the anthropomorphic characteristics of the embodied virtual body may influence participants’ behaviors, beliefs, and social and cognitive functioning in virtual reality and in real life [97]. Furthermore, body psychotherapy affects self-construction in patients with depression [98]. These results have also been supported by many studies conducted with healthy subjects [49,65,99,100,101,102]. Table 1 aims to summarize the characteristics of the reviewed studies.

## 4. Discussion and Future Directions

The present narrative review discussed the advantages and potentiality of using body ownership illusions induced through virtual reality systems for improving mental health in clinical populations presenting alterations in body perception because of their clinical condition. Today, VR is considered an advanced form of human-computer interaction that allows participants to act, communicate, and become present in an immersive computer-generated virtual environment [1,91,107]. Taking advantage of the potential of VR technology, a large number of studies have demonstrated that the sense of embodiment of a virtual body experienced during virtual BOIs can be exploited as a powerful tool for modulating some clinical disorders (e.g., motor, pain, or psychological and psychiatric disorders) by inducing changes in the patients’ internal body representation [59,61,70,108,109,110,111].

The benefits of BOIs, and more specifically virtual BOIs in clinical populations, relies on the predictive coding hypothesis, which argues that the brain maintains an internal model of the body and the space around it, i.e., the body matrix, which allows the brain to create predictions about the upcoming sensory stimuli arriving at the body and to optimally interact with the dynamic environment around the body [1,112]. Then, top-down and bottom-up multisensory processes converge into the body matrix and re-define the place of the self, inside the body, consequently modulating the internal body representation as we interact with the surrounding environment [46,113,114]. More specifically, some authors argue that the brain creates an embodied simulation of the body to effectively control and regulate the body in the world, which includes predicting people’s actions, concepts, and emotions [1]. In this line, VR experiences attempt to replicate the sensory consequences of the individual’s actions, providing them with the same scene or body representation that they can see in the real world. To achieve this, the VR system, like the brain, maintains a model (simulation) of the body and the space around it [1]. Hence, the effectiveness of virtual BOIs relies on its capability to simulate a body representation within a virtual environment while allowing the possibility to modulate the bodily experience by designing targeted virtual bodies and environments [115]. Through VR, it is possible to trick the brain’s predictive coding mechanisms, thereby inducing the sense of ownership over a virtual body and the sense of presence in the surrounded virtual environment [1]. However, some studies shown conflicting findings when inducing virtual BOIs in clinical populations presenting body image distortions. One example is the study from Tagini and colleagues [92], in which the authors observed a reduced proprioceptive drift compared to that of healthy subjects when inducing the VHI in patients with obesity. However, no differences between healthy and patients with obesity were found when inducing virtual-full BOI to investigate whether ownership over a virtual body with a skinny abdomen might be successfully experienced in patients with obesity [93]. One crucial difference between these two studies can be that one used virtual-full BOI toward a body part [92], while the other used a virtual-full BOI [93]. Such conflicting results, show that even though virtual BOIs can be a powerful tool for modifying body representation in clinical populations, a standardized methodology, or more standardized measures to assess the level of the illusion are needed to better understand which patients can benefit from them, and which not. According to this, some review studies aimed at reviewing the effectiveness of the MVFT for pain relief [116], or motor recovery following a stroke injury [117] reported a moderate quality of evidence for motor recovery and pain when using MVFT as an additional intervention. One explanation of the results obtained in the latter commented reviews could be the limitation in inducing full BOIs through mirrors, and that the perceived internal distortion of the affected body part influenced the vividness of the ownership illusion of the healthy body part reflected in the mirror. The mismatch between the internal distorted representation of the body part and the observed reflection of the body part in a normal position could reduce the feeling of ownership of the reflected body part, thus reducing the effectiveness of the therapy. In this regard, VR enables the possibility to induce embodiment of a full virtual body observed from a first-person perspective [50], and the manipulation of morphological characteristics of the represented virtual body [59,60]. Moreover, using virtual BOIs it is possible to go beyond the mirroring of movement performed with the healthy hand. Using human-machine interfaces, e.g., electromyography band reading muscle activation signals from the stump, amputees could be able to control the movement of the virtual using the amputated limb itself. Preliminary applications have shown how this type of more direct control, may foster the vividness of the illusion and in turns seems to improve the analgesic effects of the intervention [118]. A factor that has to be controlled when using virtual BOIs in patients with mental health conditions is the VR side effects such as nausea, dizziness or headaches, which may occur in some cases after wearing the head-mounted display [119]. Nevertheless, there is extensive evidence of how the fast-paced improves in VR technology is mitigating more and more this type of VR side effects.

Even though virtual BOIs effectively modulate the participants’ internal body representation by changing the morphological characteristics of the virtual body, they still present some limitations in modulating the inner world of participants, including their interoceptive, proprioceptive, and vestibular sensations. In this regard, Riva and colleagues (2017) [3] presented a new concept known as ‘sonoception’ through which it is possible to modify not only the external representation of the body but also the inner body perception [6]. Sonoception is a new noninvasive technological paradigm based on wearable acoustic and vibrotactile transducers as a new approach to modulate, augment, and replace the contents of the inner body [6]. The first aim of this approach is the development of an interoceptive simulator that can assess interoceptive time perception in clinical populations, as well as enhance heart rate variability (short-term vagally mediated component) [120], through the modulation of the parasympathetic system [121]. Similarly, others have attempted to create ‘interoceptive illusions’ by giving the participants a false acoustic feedback of their heart-rate frequency during an effortful cycling task [122]. In contrast with the full BOI, ‘interoceptive illusions’ can be induced by proving more sensory information such as vibration or acoustic feedback, instead of the visual morphological characteristics of the body. Hence, the combination of this new technological approach together with full BOIs may represent an optimal solution to fully modulate internal and external aspects of body representation in clinical populations who have an altered body perception because of their clinical condition.

## 5. Conclusions

The studies commented throughout this narrative review pave the way for the design of new rehabilitation protocols based on virtual BOIs with prolonged and repeated doses of virtual embodiment in immersive VR to tackle motor, pain, or psychological disorders, and for enabling the integration of digital technologies with existing conventional therapies. Nevertheless, virtual BOIs alone may present some limitations to fully modify the patients’ inner body perception. The new concept of ‘sonoception,’ together with virtual-full BOIs, could represent a complete solution to achieve the modulation of both the internal and the external aspects of body representation, thereby preparing the ground for a new ‘embodied medicine’ technology.

## Figures and Tables

**Table 1:** Characteristics of the reviewed studies.

Author/Date	Clinical Condition	Sample Size	Study Design	Virtual BOI (Type)	Control Condition	Multisensory Correlations (Type)	Training Period	BI and Clinical Assessment	Main Outcomes	MH Related Responses
Tagini et al., 2020 [92]	Psychiatric disorders	N = 21 patients with obesity N = 20 healthy subjects	Between-groups study	Virtual hand illusion	Async. VTS Healthy control group	Two sessions of 3 min of sync. VTS	N/A	Illusion questionnaire from (2007) [103]. Proprioceptive drift. Sensory Susceptibility Scale [104]	Findings demonstrate that individuals affected by obesity had a typical subjective experience of the illusion, while the objective effect of the illusion on self-location was reduced.	Psychological responses
Provenzano et al., 2020 [95]	Psychiatric disorders	N = 20 patients with AN N = 20 healthy controls	Between-groups study	Full virtual body illusion	3 min of async. VTS	3 min of sync. VTS and different virtual body sizes	The experiment consisted of two sessions: a pre-experimental and an experimental session with about one week break in between, in which the individualized avatars were created.	Embodiment Questionnaire VAS emotional embodiment scale VAS Similarity and Attractiveness Ratings of the Avatars	Embodiment was stronger after sync VTS in both groups, but did not reduce BID in participants with AN. The cognitive-emotional, more than the perceptual component of BID, is severely altered in AN and perspective (1PP vs. 3PP) from which a body is evaluated may play a crucial role.	Psychological responses
Scarpina et al., 2019 [93]	Psychiatric disorders	N = 15 patients with obesity N = 15 healthy subjects	Mix-model subject-design (within conditions and between groups)	Full virtual body illusion	90 s of abdomen async. VTS	90 s of abdomen sync. VTS	N/A	Embodiment Questionnaire. Body Part Size Estimation Task.	Virtual-fullBOI was efficiently induced in individuals with obesity to the same extent as in the healthy-weight individuals. Both healthy-weight individuals and individuals affected by obesity showed a reduction of the error after the synchronous, but not the asynchronous condition, with respect to the baseline.	Psychological responses
Matamala-Gomez et al., 2019 [70]	Pain disorders	CRPS (*n*=9) and PNI (*n*=10) patients were immersed in VR and the virtual arm was shown at 4 transparency levels (transparency test) and 3 sizes (size test).	Mix-model design: between-groups, one factor (groups), within-subjects (1 × 3 and 1 × 4).	Full virtual body illusion	Different virtual arm conditions: Transparency and size.	45 s of sync. VTS per condition.	N/A	Embodiment questionnaire after each virtual reality test. Pain Numeric Intensity Scale (0 = no pain to 10 = worst pain).	All 7 conditions globally decreased pain ratings by half. Increasing transparency decreased pain in CRPS but did the opposite in PNI, whereas increasing size slightly increased pain ratings only in CRPS. Embodiment in VR can decrease pain ratings of chronic arm pain, although the type of pain determines which strategy to decrease pain is most useful.	Pain responses
Osumi et al., 2019 [77]	Amputation	N= 13 patients with phantom limb and N = 6 patients with brachial plexus avulsion injury (BPA), all experiencing phantom limb pain.	Between subjects design	Virtual arm illusion MVF.	N/A	20 min of sync. VMC.	N/A	Embodiment questionnaire Bimanual circle-line coordination task (BCT). Short-Form McGill Pain Questionnaire (SF-MPQ) (0 = no pain to 3 = severe pain).	The VR-MVF rehabilitation demonstrated significant phantom limb pain alleviation, and this had a significant relationship with the restoration of phantom limb movement. VR-MVF rehabilitation led to greater alleviation of phantom limb pain among patients with brachial plexus avulsion injury compared with amputee patients.	Pain responses
Weber et al., 2019 [86]	Neurological disorders	N = 10 outpatients with chronic stroke	Pre-post within-subjects study	Virtual arm illusion.	N/A	5 min of sync. VMC per session.	12 sessions	No embodiment assessment. Fugl-Meyer Upper Extremity Scores.	There was a small improvement in mean upper limb motor recovery that did not achieve statistical significance from baseline to post-test.	Motor responses
Solcà et al. 2018 [73]	Pain disorders	N = 24 patients with CRPS N = 24 healthy controls	Crossover double-blind study	Rubber hand illusion Virtual hand illusion	Async. VTS or heartbeat-enhanced virtual reality stimulation. Healthy controls.	90 s of sync. VTS or sync. Heartbeat-enhanced virtual reality stimulation per condition.	N/A	Proprioceptive drift Ownership Illusion questionnaire Pain visual analogue scale	The primary outcome measures for pain reduction were subjective pain ratings, force strength, and heart rate variability (HRV). Heartbeat-enhanced virtual reality reduced pain ratings, improved motor limb function, and modulated a physiologic pain marker (HRV).	Pain responses
Seinfeld et al., 2018 [49]	Psychological disorders	Experimental group: N = 20 intimate partner violence offenders Control group: N = 19 healthy controls	Between-groups (one factor)	Full virtual body illusion	Control group	2 min of sync. VMC.	N/A	Embodiment questionnaire Face-Body Compound emotion recognition test	Being embodied in a female victim who suffers verbal abuse and intimidation by a male character using VR resulted in an improvement of the ability of Offenders to recognize fear in female faces, and reduced their response bias towards wrongly attributing happy emotional states to fearful facial expressions, independently of gender.	Psychological responses
Themelis and Newport, 2018 [21]	Others	N = 28 patients with painful HOA	Two-period randomized crossover design	Stretched virtual arm illusion	Real hand condition Virtual arm illusion without being stretched.	Visuo-tactile and proprioceptive manipulation. Unspecified time.	N/A	7-point Likert scale questionnaire on six statements relating to the emotional experience, perceived hand size, susceptibility, ownership, agency over the virtual arm illusion.	Stretching the hand both inside and outside of the virtual environment led to a reduction in subjective pain ratings. Virtual stretching led to changes in body perception with no changes in pressure pain threshold.	Pain responses
Pozeg et al., 2017 [74]	Neurological disorders	N = 20 patients with paraplegia (SCI); N = 20 healthy controls	2 factorial, randomized, repeated-measures design.	Virtual leg illusion	Async. VTS.	60 s of async. VTS	N/A	9-item questionnaire adapted from body illusion studies, with items referring to the experienced ownership of the virtual legs, illusory touch, and referred touch. Pain-visual analogue scale (0 = no pain/100 = worst pain). Cambridge Depersonalization Scale (CDS) [105].	Patients with SCI are less sensitive to multisensory stimulations inducing illusory leg ownership (as compared to HC) and leg ownership decreased with time since SCI. In contrast, no differences between groups in global body ownership as tested with the FBI were found. VLI and FBI were both associated with mild analgesia that was only during the VLI specific for synchronous visuo-tactile stimulation and the lower back position.	Pain and embodiment responses
Pamment et al., 2017 [75]	Pain disorders	N = 18 chronic pain patients.	1 × 3 within-subjects study	Full virtual body illusion	Async. VTS.	2 min of sync. VTS.	N/A	7-point Likert scale embodiment questionnaire Gill Pain Questionnaire	Pain intensity in chronic pain patients was reduced by 37% by ‘out of body’ illusions.	Pain responses
Falconer et al., 2016 [96]	Psychiatric disorders	N = 15 depressive patients	1 × 2 within-subjects study	Full child virtual body illusion. Full adult virtual body illusion.	No control group/conditions	2 min of sync. VMC.	N/A	Ownership questionnaire. Patient Health Question-naire-9 (PHQ-9). Zung Self-Rating Depression Scale (SDS). Self-Compassion and Self-Criticism Scale (SCCS).	Significant reductions in depression severity and self-criticism, as well as a significant increase in self-compassion were found, from baseline to 4-week follow-up.	Psychological responses
Keizer et al., 2016 [94]	Psychiatric disorders	N = 30 AN patient	1 × 2 Mix-model design. Factor: Congruency	Full virtual body illusion	Healthy controls and async. VTS	90 s of sync. VTS per condition	N/A	Embodiment questionnaire Body Attitude Test (BAT) Eating Disorder Inventory-II (EDI-II)	It is possible to decrease AN patients’ overestimation of body size in an experimental FBI setting, with effects remaining at least up to ~2 h and 45 min after the illusion is induced.	Psychological responses
Ortiz-Catalan et al., 2016 [78]	Amputation	N = 14 amputee patients with phantom limb pain	A Single Group Clinical Trial study	Virtual arm illusion	N/A	15 min of sync. VMC.	N/A	No embodiment assessment. Numeric rating scale (0 = no pain to 10 = worst pain). Short-form McGill Pain Questionnaire.	Phantom limb pain decreased from pre-treatment to the last treatment session by 47% for weighted pain distribution, 32% for the numeric rating scale, and 51% for the pain rating index. The numeric rating scale score for intrusion of phantom limb pain in activities of daily living and sleep was reduced by 43% and 61%, respectively.	Pain responses
In et al., 2016 [88]	Neurological disorders	Experimental group (VRRT): N = 13 patients with chronic stroke Control group: N = 12 patients with chronic stroke	Between-groups study	VRRT virtual reality reflection therapy	No VRRT control group	30 min of sync VMC	4 weeks.	No embodiment assessment. Berg Balance Scale (BBS). Functional Reaching Test (FRT). Timed Up and Go (TUG).	In the change of BBS scores, both the VRRT and the control group displayed significant improvements after the intervention, and the improvement was significantly better in the VRRT group than in the control group. FRT, TUG, and 10 m Walk Test improved more in the VRRT group than in the control group.	Motor responses
Hwang et al., 2014 [80]	Pain disorders	Experimental group (Virtual Body Swapping with mental rehearsal) N = 13 CRPS patients. Control group1 (Mental rehearsal): N = 13 CRPS patients. Control group2 (Watching movement): N = 13 CRPS patients.	Within-subjects study	Body swapping	Control groups 1 and 2	Experimental group: 1PP Motor imagery VMC + virtual arm VMC Control group 1: 1PP Motor imagery VMC. Control group 2: Real arm VMC. Unspecified time.	N/A	Illusion strength questionnaire. Body distortion questionnaire [106]. Pain Intensity questionnaire.	Pain intensity did not decrease significantly after treatment in any of the groups. Body Perception Disturbance improved significantly after treatment in the VBS group, but not in the other groups.	Pain responses
Alphonso et al. 2012 [79]	Amputation	N = 18 patients with trans-radial/trans-humeral amputation	Within-subjects study	Virtual arm illusion	No control condition	Two 10 min of sync. VMC, with a break of 5 min.	20 days.	No embodiment assessment. 100 mm- visual analogue scale (0 = no pain to 100 = worst pain).	Data from the visual analogue scale showed a decrease in phantom limb pain as the virtual integrated environment sessions increased.	Pain responses

Sync: synchronous; Async: asynchronous; VTS: Visuo-tactile stimulation; VMC: Visuo-motor correlations; N/A: Not applicable; CRPS: Complex Regional Pain Syndrome; PNI: Peripheral Nerve Injury; BPA: Brachial Plexus Avulsion; HC: Healthy Control; HOA: Hand Osteoarthritis; MVF: Mirror Visual Feedback; VBS: Virtual Body Swapping; VRRT: Virtual Reality Reflection Therapy; 1PP: First Person Perspective; 3PP: Third Person Perspective; VAS: Visual analogue Scale; BID: Body image distortion; SCI: Spinal cord Injury; HC: Healthy Control; FBI: Full-Body Illusion; VLI: Virtual Limb Illusion.
